# Puerarin alleviates inflammation and pathological damage in colitis mice by regulating metabolism and gut microbiota

**DOI:** 10.3389/fmicb.2023.1279029

**Published:** 2023-10-16

**Authors:** Yixin Zou, Wenjiao Ding, You Wu, Tingting Chen, Zheng Ruan

**Affiliations:** State Key Laboratory of Food Science and Resources, School of Food Science, Nanchang University, Nanchang, China

**Keywords:** puerarin, ulcerative colitis, network pharmacology, gut microbiota, untargeted metabonomics

## Abstract

Dysbiosis of gut microbiota and metabolic pathway disorders are closely related to the ulcerative colitis. Through network pharmacology, we found that puerarin is a potential ingredient that can improve the crypt deformation and inflammatory infiltration in mice, and decrease the levels of IL-1β, IL-6 and TNF-α significantly. *Listeria*, *Alistipes* and *P. copri* gradually became dominant bacteria in UC mice, which were positively correlated with inflammatory factors. Puerarin effectively improved dysbiosis by reducing the abundance of *Alistipes*, *P. copri* and *Veillonella*, and increasing the level of *Desulfovibrionacea*. Correlation network and metabolic function prediction analysis of the microbiota showed that they formed a tightly connected network and were widely involved in carbohydrate metabolism and amino acid metabolism. Specifically, we observed significant changes in the tryptophan metabolism pathway in DSS mice, with an increase in the abundance of *Bacteroidetes* and *Enterobacteriaceae* involved in tryptophan metabolism. However, this metabolic disorder was alleviated after puerarin treatment, including the reversal of 3-HAA levels and an increase in the abundance of *Rhodobacteraceae* and *Halomonadaceae* involved in kynurenine metabolism, as well as a significant increase in the purine metabolite guanosine. In conclusion, our study suggests that puerarin has a good therapeutic effect on UC, which is partially achieved by restoring the composition and abundance of gut microbiota and their metabolism.

## 1. Introduction

Ulcerative colitis (UC) is a relapsing disease characterized by chronic inflammation. Clinical symptoms include diarrhea, abdominal pain, and hematochezia. According to the latest data, the prevalence of UC was estimated to be 5 million cases around the world, and there is an increasing trend in some emerging industrialized countries (Le Berre et al., 2023; Liu J. et al., 2023).

The pathogenesis of UC is multifactorial, of which the epithelial barrier defects and disorder of immune response are important factors. Persistent inflammation in the gut can lead to dysbiosis of gut microbiota and abnormal accumulation of microbiota metabolites. These abnormal metabolites can further stimulate the immune system, ultimately resulting in impaired mucosal barrier function and exacerbated inflammation (Neurath, 2014).

The current mainstream treatment for UC is the use of salicylic acid drugs to inhibit the synthesis of prostaglandins and leukotrienes. However, they have significant side effects, such as nausea, rash and liver dysfunction, which make them unsuitable for long-term use (Kucharzik et al., 2020). Given the long course and high recurrence rate of UC, it is necessary to search other natural active substances for intervention to achieve a relieving effect.

*Radix Puerariae* is the dried root of the leguminous plant *Pueraria lobata*, which contains abundant isoflavones and has been widely used in the food and health product industries (Wang et al., 2022). Previous studies have found that water extracts containing *Radix Puerariae* can increase tight junction protein expression (Wang et al., 2023), lower NF-κB activation, reduce inflammatory factor expression in the colon and relieve oxidative stress (Li et al., 2016). These findings suggest that *Radix Puerariae* has a beneficial regulatory effect on intestinal barrier function and inflammation. However, its specific active ingredients and their effects on the composition of the intestinal microbiome and metabolite levels in UC have not been fully elucidated.

In recent years, with the proposal of disease network and systems pharmacology theory (Nogales et al., 2022), it has become a convenient and efficient method to systematically analyze and predict the “substance-gene-target-disease” action network and find potential effective active ingredients based on technologies such as genomics, high-throughput screening, network visualization, and network analysis (Wang et al., 2021).

Based on this, this article first uses network pharmacology to mine the single active ingredients in *Radix Puerariae*, and then establishes a DSS-induced colitis mouse model to verify its improvement effect. In order to further explore possible mechanisms of action, untargeted metabolomics and 16S rRNA technology are used to investigate the improvement of gut microbiota and metabolites by active ingredients.

## 2. Materials and methods

### 2.1. Virtual compound screening

We utilized the TCMSP database^1^ to retrieve compounds by searching for ‘*Radix Puerariae*’ as the primary keywords. To screen the active ingredients and corresponding targets, we applied OB ≥ 30% and DL ≥ 0.18 as screening conditions (Jiao et al., 2022). Additionally, we included substances with active ingredients that have been widely reported in *Radix Puerariae* for subsequent analysis. Then combined these active targets with compound active targets obtained from the Drugbank database^2^ to obtain the total active targets for pueraria. Subsequently, we used uniprot database^3^ to convert the corresponding gene names (Zhang H. et al., 2021).

For searching the disease targets, we used DisGeNET^4^ and GeneCards database.^5^ Then used Cytoscape software to map the cross-gene information between UC and *Radix Puerariae*. Additionally, we conducted PPI analysis using the STRING database,^6^ as well as GO and KEGG analyses using the DAVID database^7^ (Li et al., 2022).

### 2.2. Molecular docking

We searched the PubChem database^8^ to obtain the 3D structure files of puerarin, genistein, formononetin, and daidzein. And downloaded the crystal structures of TNF-α, IL-1B, and IL-10 from the PDB database.^9^ We then docked the small molecules with these proteins and visualized the results of small molecule-protein binding pockets and interactions between the small molecules and amino acid residues by using PyMol 2.5.5 software.

### 2.3. Animals and experimental treatment

C57BL/6J mice (male, 6–7 weeks old, 20–23 g) were obtained from Spafu Biotechnology (Beijing, China) and housed in a SPF animal facility with a 12 h light/dark cycle. The mice were provided with standard rodent feed and purified water, and the ambient relative humidity was maintained between 40 and 60% at a temperature of 25°C ± 2°C. The experiment was approved by the Animal Experimentation Ethical Committee of Nanchang University (License number: SYXK 2021-0001), and all animal feeding and procedures were conducted in accordance with the experimental animal welfare ethical code of Nanchang University.

**Figure 1:**
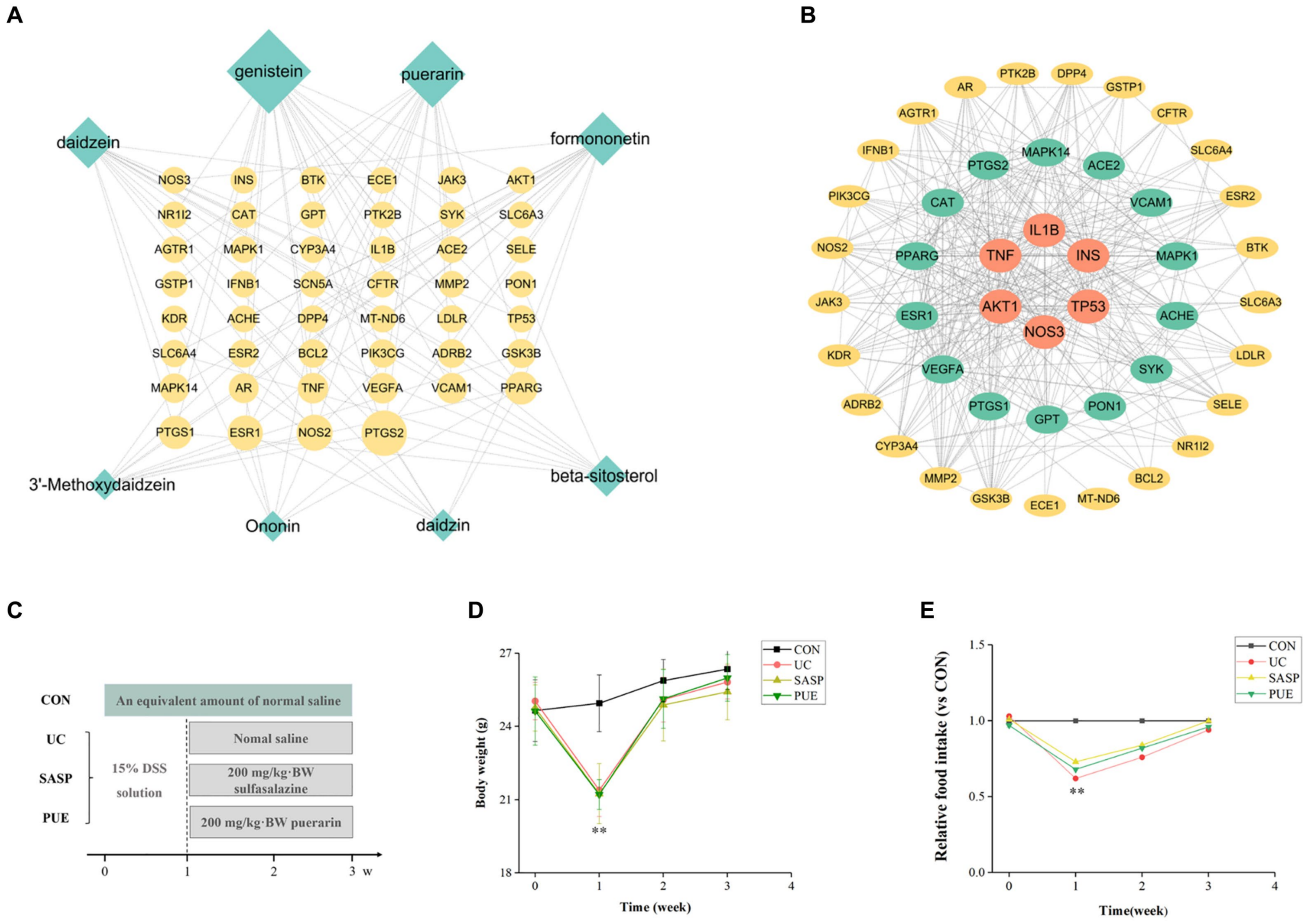
Active ingredient puerarin was screened out by network pharmacology, and it has a good effect on the growth performance of mice. **(A)** Diagram of overlapping genes’ network between UC and active components in *Radix Puerariae*. **(B)** Protein–protein interaction analysis of overlapping genes. **(C)** Schematic diagram of mouse modeling. **(D)** Changes in body weight of mice during the experiment. **(E)** Changes of food intake of mice compared with CON group during the experiment. ^**^p < 0.01, compared to the CON group.

The animal experiment procedures are depicted in Figure 1C. After 1 week of adaptive feeding, mice were randomly assigned to four groups: Control group (CON), Model group (UC), Positive group (Sulfasalazine, SASP), and Puerarin group (PUE). All mice were fed the same diet. During the first week, the Model group, Positive group, and Puerarin group received intragastric administration of DSS aqueous solution three times a day, while the Control group received an equivalent amount of normal saline. For the next 2 weeks, the Puerarin group received 200 mg/kg·BW puerarin via gavage, while the Positive group received 200 mg/kg·BW SASP via gavage (Huang et al., 2017). The Model and Control groups were administered an equivalent volume of normal saline via gavage. The weight and diet of all mice were monitored on a weekly basis.

### 2.4. Colitis induction and evaluation

To ensure that each mouse receives an equal dose of DSS during modeling, we induced colitis by gavage with DSS in this study. During the first week, mice in the model group were gavaged with 0.1 mL/g (0.23 mL) of 15% DSS water solution at 10:00, 15:00 and 20:00 each day until the end of modeling, with an average cumulative intake of DSS of 31.5 mg/g per mouse. After modeling, the DAI index was used for preliminary evaluation of the model (Peng et al., 2020). The detailed scoring criteria can be found in Supplementary Table S1.

### 2.5. Biochemical assays of the serum

Whole blood was obtained by puncturing the submandibular vein. To be specific, we inserted a blood collection needle into the posterior aspect of the top of the mandible, where the vein was located, and collected blood. After that, we applied a dry cotton ball to the puncture site to stop bleeding. The whole blood of mice was placed in the tube and centrifuged at 3000 rpm for 15 min at 4°C to obtain serum. The concentrations of TNF-α, IL-6, and IL-1β in the serum were measured using an ELISA kit in accordance with the manufacturer’s instructions (Zhao et al., 2021).

### 2.6. Hematoxylin and eosin staining

The colon tissue was fixed in 4% paraformaldehyde, embedded in paraffin to make 4 mm thick sections, stained with hematoxylin and eosin (H&E) staining solution, and observed under the orthostatic optical microscope (Zhang et al., 2022).

### 2.7. 16S rRNA and microbiota data analysis

We used QIAamp DNA Stool Kit (Qiagen, Hilden, Germany) and extracted the DNA according to the instructions. The V3-V4 region of the gene was amplified using primers with barcodes, and the resulting amplicons were sequenced on the Illumina NovaSeq platform (Yan et al., 2020).

The initial sequencing data were processed using QIIME2 (2021.8) to generate a sample metadata file (Ding et al., 2021). DADA2 was used for denoising analysis to obtain corresponding feature tables and ASVs data. A Venn diagram was constructed to display the diversity information of ASVs data, showing the abundance of different species and groups. Beta diversity analysis was performed to reveal differences in community structure among different sample groups using principal coordinates analysis (PCoA) and orthogonal partial least squares discriminant analysis (OPLS-DA). Linear discriminant analysis effect size (LEfSe) was used to identify significant differences in microbial composition between different groups (Xu et al., 2022).

### 2.8. Untargeted metabolomics

In untargeted metabolomics experiments, an X500R HPLC-Q-TOF/MS (SCIEX) was used with an XSelect Premier HSS T3 column (100 mm × 2.1 mm, Waters Corporation, Milford, Massachusetts, USA) for chromatographic separation (Jing et al., 2021). The mobile phase consisted of 0.1% formic acid in water (solvent A) and acetonitrile (solvent B), with a gradient duration of 18 min and a flow rate of 0.3 mL/min (Mi et al., 2023). ESI was used for ionization in both positive and negative ion modes, with ion source gasses 1 and 2 set at 55 psi and curtain gas at 35 psi. The ion source temperature was set to 550 C, while the positive and negative ion source voltages were set to 5,000 V and − 4,500 V, respectively. TOF/MS scans were performed over a m/z range of 50–1,200 Da, while TOF/MS/MS scans were performed over a m/z range of 30–1,200 Da. MS/MS data was detected using information-dependent acquisition (IDA).

Data acquisition was controlled using SCIEX OS 2.0 software (AB SCIEX). Metabolite annotation was performed using MetDNA and SCIEX OS 2.0 software. Statistical analysis was conducted using unsupervised principal component analysis (PCA) and supervised orthogonal partial least squares discriminant analysis (OPLS-DA) in MetaboAnalyst 5.0^10^ to identify differences in the studied metabolites and screen for potential biomarkers (Zhang Q. et al., 2021). After verifying the differential metabolites in the public database HMDB, we performed metabolic pathway analysis based on the KEGG database (Zhou et al., 2021).

### 2.9. Statistical analysis

The data collected in this study were analyzed by using SPSS statistical software (24.0). One-way analysis of variance (ANOVA) was used to compare mean differences among the groups, with significance set at *p* < 0.05. The results were expressed as mean ± SD.

## 3. Results

### 3.1. Puerarin is a potential ingredient that restored the growth performance of colitis mice

A total of 8 potential active components have been screened from *Radix Puerariae* (detailed information can be found in Supplementary Table S2) and identified 46 overlapping genes between core disease targets (1090) and active ingredient targets (113). Using Cytoscape software, we constructed a network diagram of the ingredient-disease-target interactions (Figure 1A), which revealed that genistein, puerarin, and daidzein had the highest degree of overlap with disease targets. We also performed protein interaction analysis on the overlapping genes and identified key genes such as IL1β, TNF, TP53, and INS (Figure 1B), and are involved in TNF, PI3K-Akt, and VEGF signaling pathways (Supplementary Figures S1–S3).

**Figure 2:**
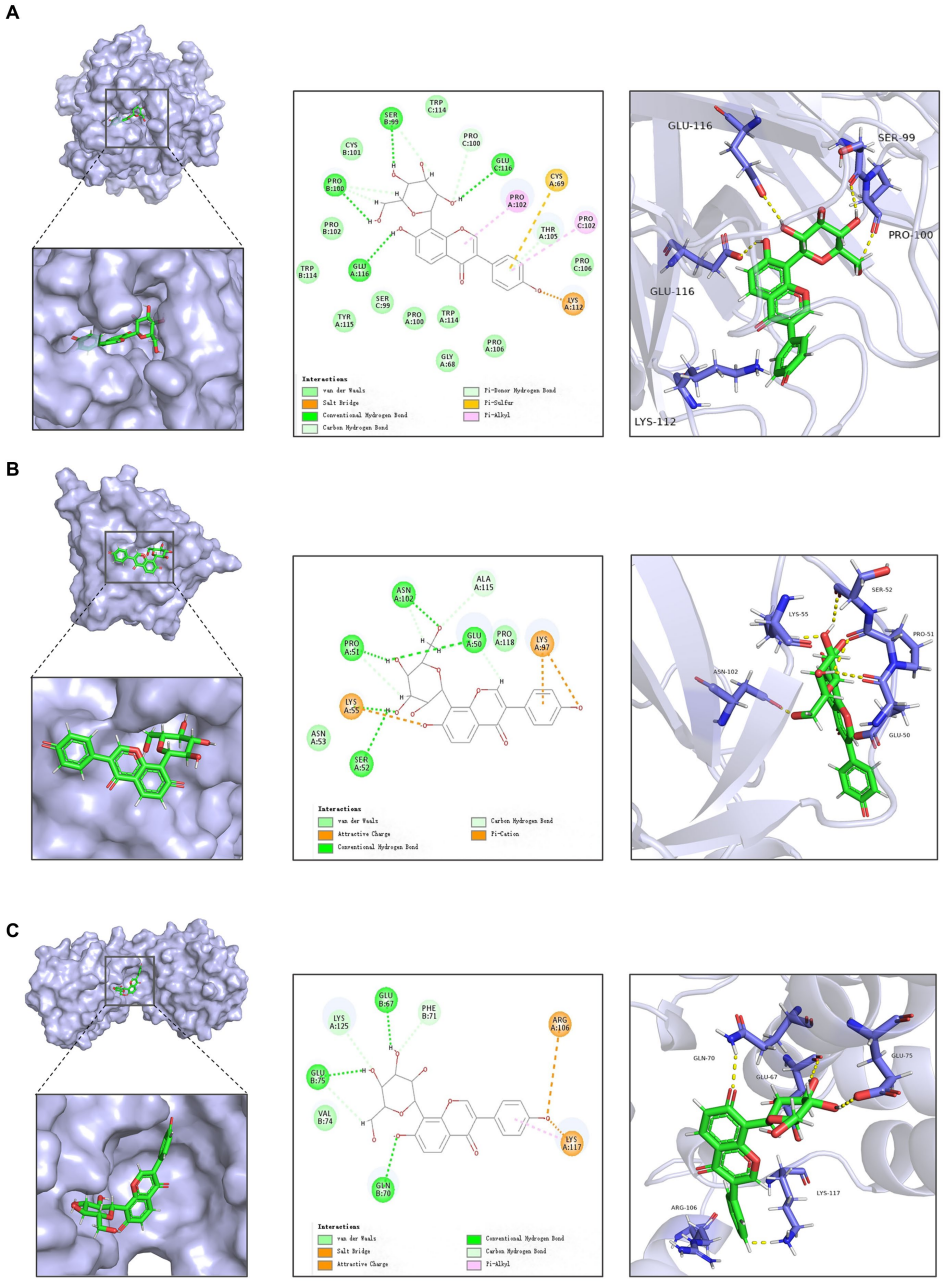
Docking of active ingredients in *Radix Puerariae* with target molecules. **(A)** Schematic diagram of docking of puerarin with TNF-α. **(B)** Schematic diagram of docking of Puerarin with IL1B. **(C)** Schematic diagram of docking of Puerarin with IL10.

Subsequent molecular docking revealed that puerarin had the most stable docking with the core target (Figure 2; Supplementary Table S3). And considering the low OB and DL values of genistein (OB = 17.93%, DL = 0.21), we prioritized the use of puerarin, which showed similar good effect, for subsequent animal experiments.

As shown in Figures 1D,E, mice that were orally administered DSS in the first week exhibited a significant decrease in body weight and food intake compared to the control group (*p* < 0.01). However, after stopping the modeling and supplementing with puerarin or sulfasalazine, the body weight of mice gradually recovered, and their food intake also increased. After 2 weeks of oral administration, the food intake of mice in the modeling group gradually returned to normal levels and approached those of mice in the control group.

### 3.2. Puerarin alleviates the symptoms of DSS induced colitis in mice

**Figure 3:**
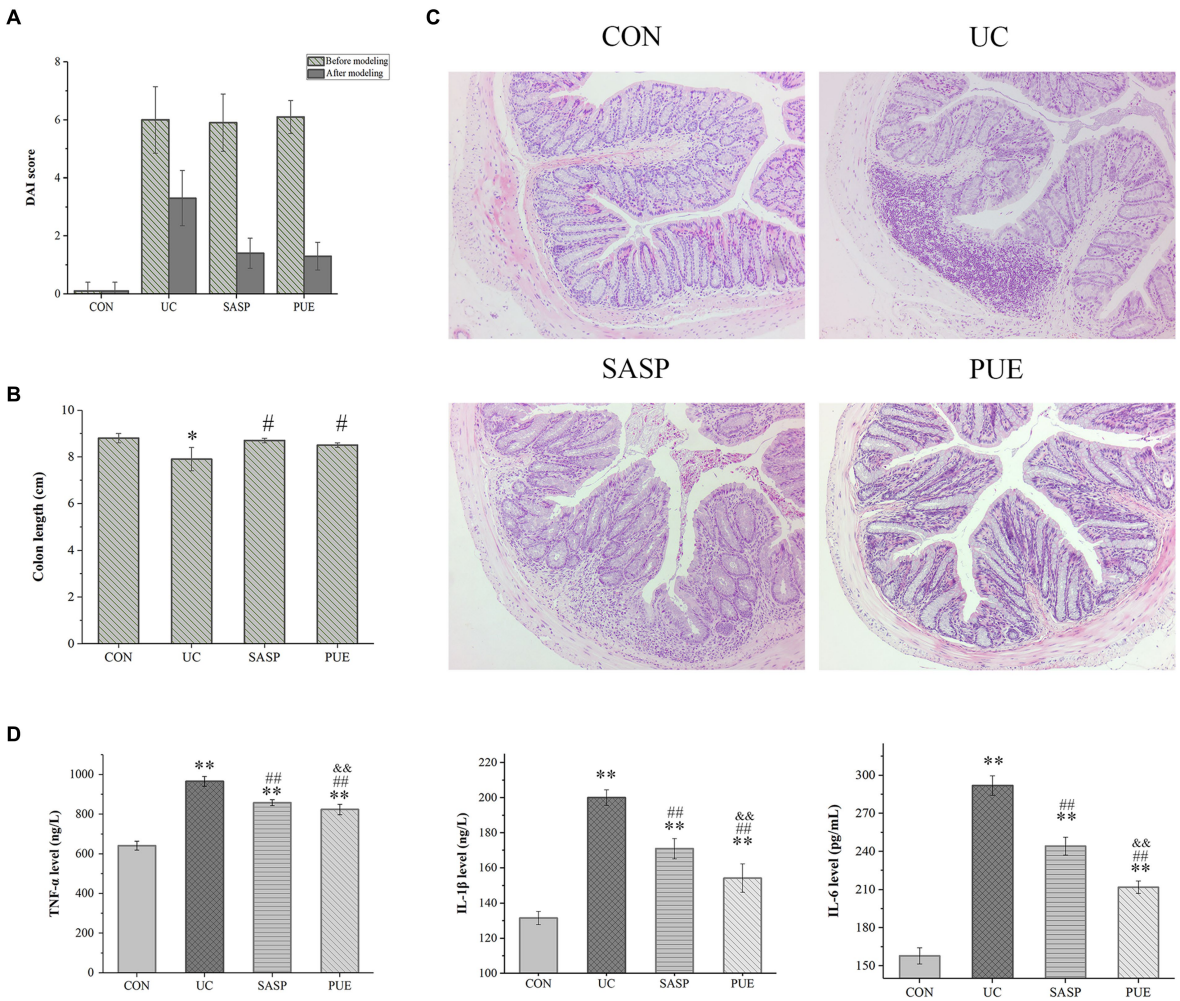
Puerarin improved disease score, colonic tissue morphology and reduced inflammatory factor expression in mice. **(A)** DAI score of each group at the end of modeling and administration. **(B)** Colon length of mice in each group. ^*^p < 0.05, compared to the CON group; ^#^*p* < 0.05, compared to the UC group. **(C)** HE section of colon of mice in each group. **(D)** The expression levels of IL-1β, IL-6 and TNF-α in each group (*n* = 8). ^**^p < 0.01, compared to the CON group; ^##^*p* < 0.01, compared to the UC group; ^&&^*p* < 0.01, compared to the SASP group.

We performed DAI scoring on mice at the end of both modeling and treatment (Figure 3A). Representative images of fecal samples from mice at the end of modeling/treatment are shown in Supplementary Figure S4A. The control group showed no abnormalities throughout the experiment, while severe bloody stools were observed in the other three groups after modeling. Some mice in the UC group still had mild red stool, while some mice in the PUE group had mild soft stools without blood and had returned to normal status.

Observation of colon tissue morphology revealed that DSS-induced colitis significantly shortened colon length (*p* < 0.05), while supplementation with puerarin significantly increased colon length compared to the UC group (*p* < 0.05), and showed no difference compared to the control and SASP groups (Figure 3B; Supplementary Figure S4B).

### 3.3. Puerarin improved colonic tissue morphology and reduced inflammatory factor expression

Figure 3C shows the HE staining of intestinal sections from mice in each group. In the control group, the colon tissue appeared normal, with intact crypt epithelial cells, goblet cells, and submucosa. However, in the UC group induced by DSS, ulcers appeared in the colon and spread to the mucosa and submucosa. The mucosal layer showed infiltration of inflammatory cells, and there was significant damage to the crypts with structural deformation. In contrast, mice treated with PUE did not show obvious colonic ulcers, and only a small amount of inflammatory cell infiltration was observed in the mucosal layer. The deformation of crypts also improved.

As shown in Figure 3D, compared to the CON group, the concentrations of IL-1β, IL-6 and TNF-α were significantly increased (*p* < 0.01) in the UC group. This suggests that there was significant inflammation in the intestines of UC mice. After treatment with SASP or PUE, the concentrations of these three cytokines significantly decreased (*p* < 0.01), but did not fully recover to normal levels (vs CON). Notably, PUE showed a more significant decrease in IL-1β and IL-6 compared to SASP, indicating that puerarin has a better effect on improving inflammation caused by DSS-induced cytokine surge.

### 3.4. Effect of puerarin on intestinal microorganisms in mice with colitis

**Figure 4:**
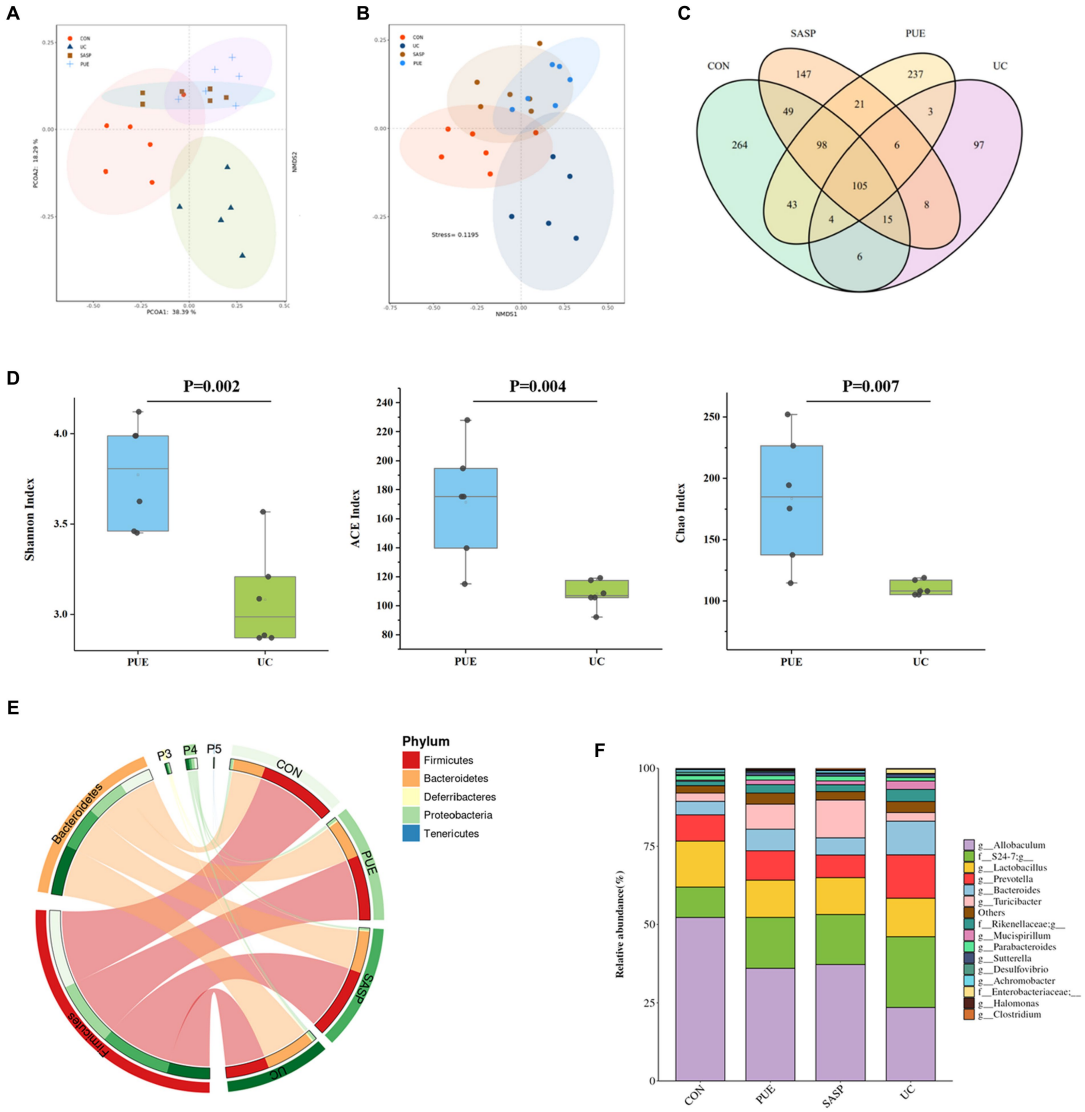
Changes of microbiota composition in mice after puerarin intervention. **(A)** PcoA diagram. **(B)** NMDS chart. **(C)** ASV Venn diagram of mice in each group. **(D)** Comparison of α diversity between UC and PUE groups. **(E)** Species composition at phylum level. **(F)** Species composition at genus level.

By examining the species accumulation curve in microbial analysis (Supplementary Figure S5) and conducting α-diversity, β-diversity analysis on different microbial groups, the sample size and community richness were evaluated. The PCoA (Figure 4A) and NMDS plot (Figure 4B) showed significant separation between the UC group and the control group, indicating that DSS significantly altered the composition of the microbiota in mice, which was distinct from that of the control group. Moreover, there was no overlap between the PUE group, SASP group and UC group, suggesting that administration induced significant changes in gut microbiota composition with a trend toward normalizing to that of the control group.

A Venn diagram was constructed to characterize the overlap of ASVs among the microbiota based on the ASV information table obtained from QIIME2 denoising analysis. As shown in Figure 4C, the four groups contained 264 (CON), 97 (UC), 147 (SASP), and 237 (PUE) unique ASVs respectively, with a total of 105 ASVs shared as their core microbiota. The results indicated that induced colitis significantly reduced gut species composition, while the number of ASVs increased after SASP intervention and was significantly higher after PUE intervention.

Figure 4D displays the changes in α-diversity of gut microbiota after modeling and intervention. It was observed that the Shannon, ACE, and Chao indices of mice in the UC group were significantly lower than those in the PUE group, indicating that puerarin intervention partially restored the species composition and abundance of gut microbiota in mice.

Furthermore, the top 5 species at the phylum level and top 10 species at the genus level were analyzed (Figures 4E,F). The major microbiota included *Firmicutes*, *Bacteroidetes*, *Proteobacteria*, *Deferribacteres* etc. DSS modeling increased the level of *Bacteroidetes* and decreased the level of *Firmicutes*. However, compared to the UC group, the PUE group reversed the change in F/B ratio and showed a trend toward normalization to that of the control group.

The major species at the genus level included *Allobaculum*, S24-7, *Lactobacillus*, *Prevotella*, *Bacteroides*, and *Turicibacter*. Comparing the changes in gut microbiota at the genus level revealed that DSS modeling significantly increased the levels of *Prevotella*, S24-7, *Bacteroides* and *Mucispirillum*, while decreased the levels of *Allobaculum* and *Lactobacillus*. Compared to the UC group, PUE intervention reversed the changes in *Allobaculum*, *Prevotella*, *Bacteroides*, and *Mucispirillum* levels while decreased S24-7 levels and moving toward a microbiota structure similar to that of the control group.

**Figure 5:**
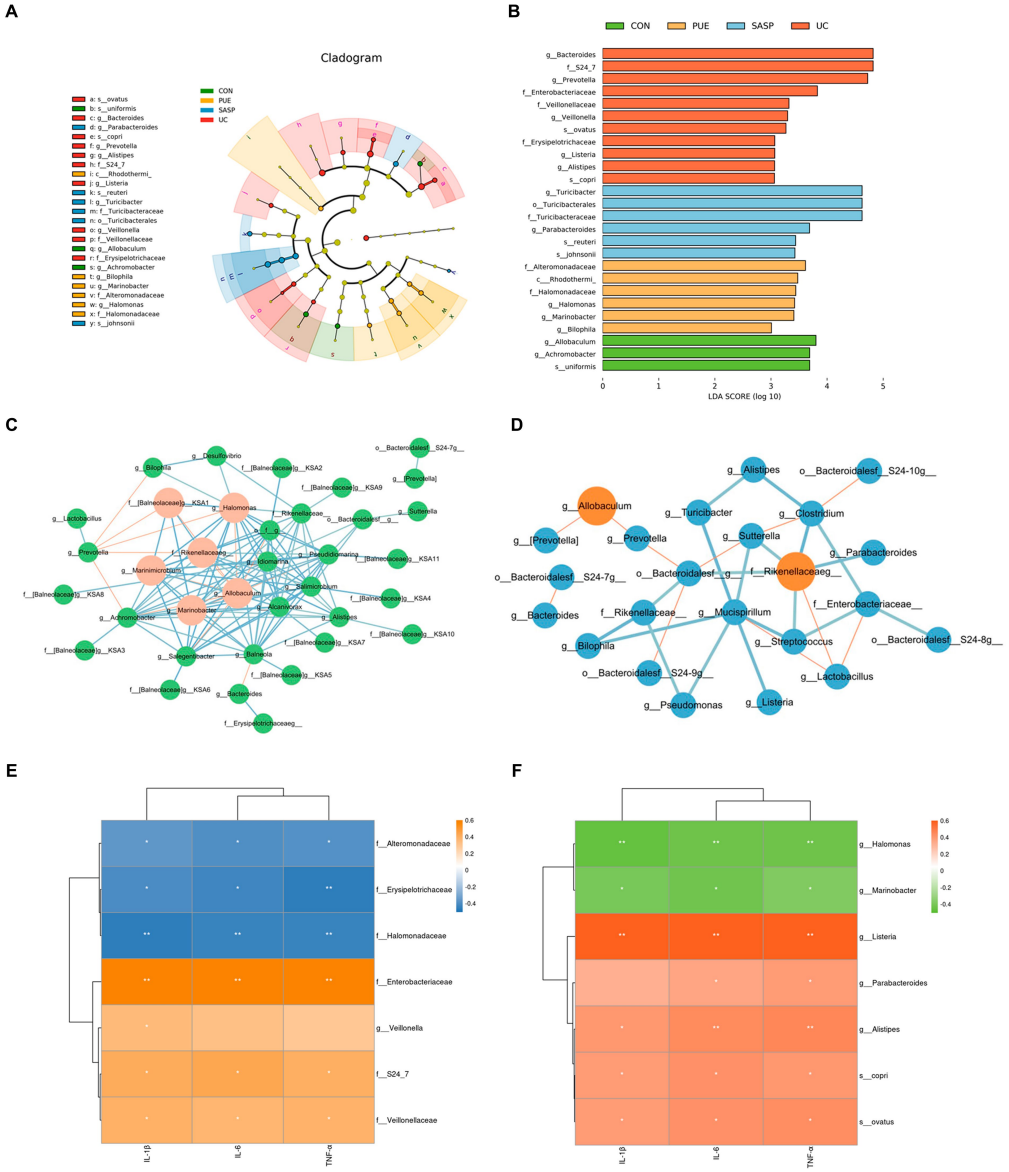
**(A)** Evolutionary branching diagram. **(B)** LEfSe multi-level species discrimination. **(C)** Correlation network of gut microbiota in PUE Group. **(D)** Correlation network of gut microbiota in UC Group. The red lines indicate negative correlation, while the blue lines indicate positive correlation. The red circles represent the bacteria with a larger number of associations. **(E,F)** The heat map of correlation analysis between inflammatory factors and significantly different microorganisms. ^*^ indicates *p* < 0.05; ^**^ indicates *p* < 0.01.

In addition, we used linear discriminant analysis effect size (LEfSe) to examine the differences in fecal species abundance between two groups of mice at different levels (Figures 5A,B). A total of 26 differentially abundant species were identified, including 11 in the UC group: *Prevotella*, S24-7, *Bacteroides*, *Veillonella*, *Veillonellaceae*, *Listeria*, *Eysipelotrichaceae*, *Ovatus*, *Alistipes*, *Copri* and *Enterobacteriaceae*. The PUE group had 6 differentially abundant species: *Rhodothermi*, *Alteromonadaceae*, *Halomonas*, *Halomonadaceae*, *Marinobacter* and *Bilophila*. The SASP intervention had 6 significant species: *Turicibacter*, *Turicibacteraceae*, *Turicibacterales*, *Reuteri*, *Parabacteroides*, and *Johnsonii*.

In order to further elucidate the potential relationships among gut microbiota, we conducted a correlation analysis of the gut microbiota in UC and PUE mice. The resulting visualization network diagram is shown in Figures 5C,D. Our analysis revealed that in the UC state, the abundance of *Allobaculum*, which is notably reduced in the UC state, exhibits a significant negative correlation with the dominant bacterium *Prevotella*. While various pathogenic bacteria such as *Alistipes*, *Listeria*, and *Rikenellaceaeg* formed a tightly connected network with multiple bacteria. After puerarin intervention, we observed a significant enrichment of *Halomonas*, while *Bilophila* and *Halomonas* were negatively correlated with *Prevotella*. Additionally, *Allobaculum* was negatively correlated with *Marinobacter* and *Marinimicrobium*. These correlation analysis results indicate that there are extensive connections among gut microbiota, and the dominance of different bacteria in different states may be due to their interactions with each other.

Similarly, to understand the relationship between differentially abundant species and inflammatory factors, we conducted a correlation analysis between inflammatory factors and significantly different species (Figures 5E,F). We found that in the UC group, *Enterobacteriaceae*, *Alistipes* and *Listeria* (*p* < 0.01), *Copri*, *Veillonellaceae* and S24_7 (*p* < 0.05) were positively correlated with the concentration of pro-inflammatory factors, while *Eysipelotrichaceae* (*p* < 0.01) was negatively correlated with them. In the PUE group, *Alteromonadaceae* and *Halomonadaceae* (*p* < 0.05) were negatively correlated with the concentration of pro-inflammatory factors, while *Balneola* and *Marinobacter* were negatively correlated with TNF-α (*p* < 0.05).

### 3.5. Effect of puerarin on intestinal metabolism in mice with colitis

**Figure 6:**
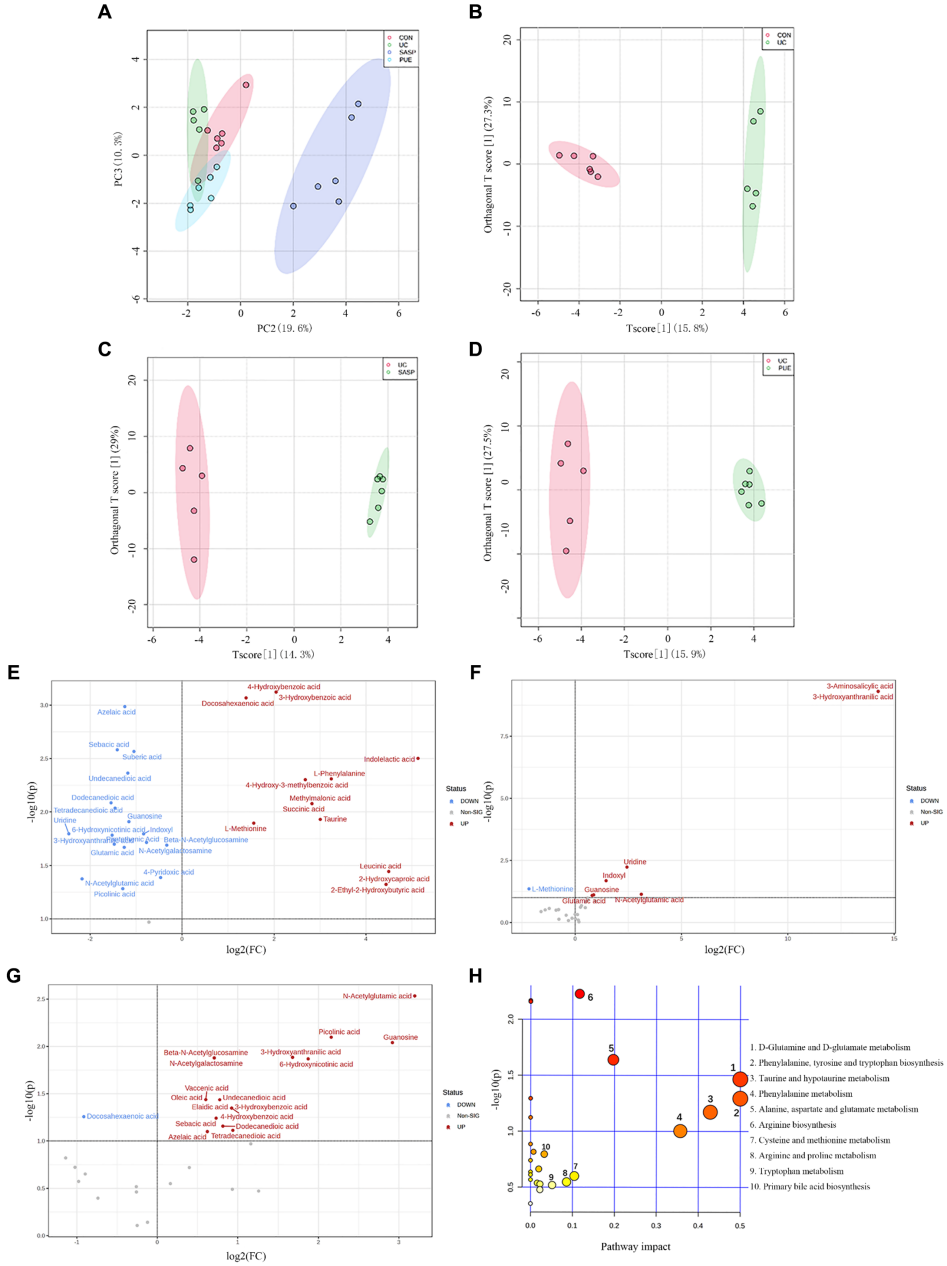
Changes of metabolite levels in each group. **(A)** PCA diagram between four groups. **(B–D)** OPLS-DA diagram between two groups. CON vs. UC; UC vs. SASP; UC vs. PUE. **(E)** Volcano plot of untargeted differential metabolites in UC mice compared to control group. **(F)** Volcano plot of differential metabolites in SASP mice compared to UC group. **(G)** Volcano plot of differential metabolites in PUE mice compared to UC group. **(H)** Bubble plot of differential metabolic pathways analysis in UC mice compared to control group.

In unsupervised mode (Figure 6A), the UC group was clearly separated from the control group, indicating significant differences in fecal metabolites between the two groups; PUE and SASP interventions caused their metabolite characteristics to move away from the UC group and toward the control group. At the same time, in the supervised OPLS-DA mode, each group could also be well separated (Figures 6B–D): UC vs. control group (R2 = 0.618, Q2 = 0.342), SASP vs. UC group (R2 = 0.793, Q2 = 0.364), PUE vs. UC group (R2 = 0.602, Q2 = 0.35). The results indicate that there are differences in metabolites in feces of each group.

By setting the conditions of FC >1 or FC <1/2 and *p* < 0.1, differentially abundant metabolites were screened out, and volcano plots were used to represent the differentially abundant metabolites obtained from different groups (Figures 6E–G). From the figure, it can be seen that compared to the CON group, there were 13 metabolites (such as 3-hydroxybenzoic acid, docosahexaenoic acid, indolelactic acid, etc.) increased and 18 metabolites decreased (such as azelaic acid, sebacic acid, uridine, etc.) in the UC group. Compared to the UC group, there were 7 metabolites increased and 1 metabolite (L-methionine) decreased in the SASP group; while there were 17 metabolites increased and 1 metabolite (docosahexaenoic acid) decreased in the PUE group. Specific information on differentially abundant metabolites can be found in Supplementary Tables S4–S6.

Further setting VIP value >1, 26 differentially abundant metabolites obtained from the secondary screening of the UC group were subjected to metabolic pathway analysis (Figure 6H), and it was found that these metabolites had a major impact on 7 metabolic pathways, including the biosynthesis of phenylalanine, tyrosine and tryptophan, D-glutamine and D-glutamate metabolism, taurine and hypotaurine metabolism, phenylalanine metabolism, alanine, aspartate and glutamate metabolism, arginine biosynthesis and cysteine and methionine metabolism.

**Figure 7:**
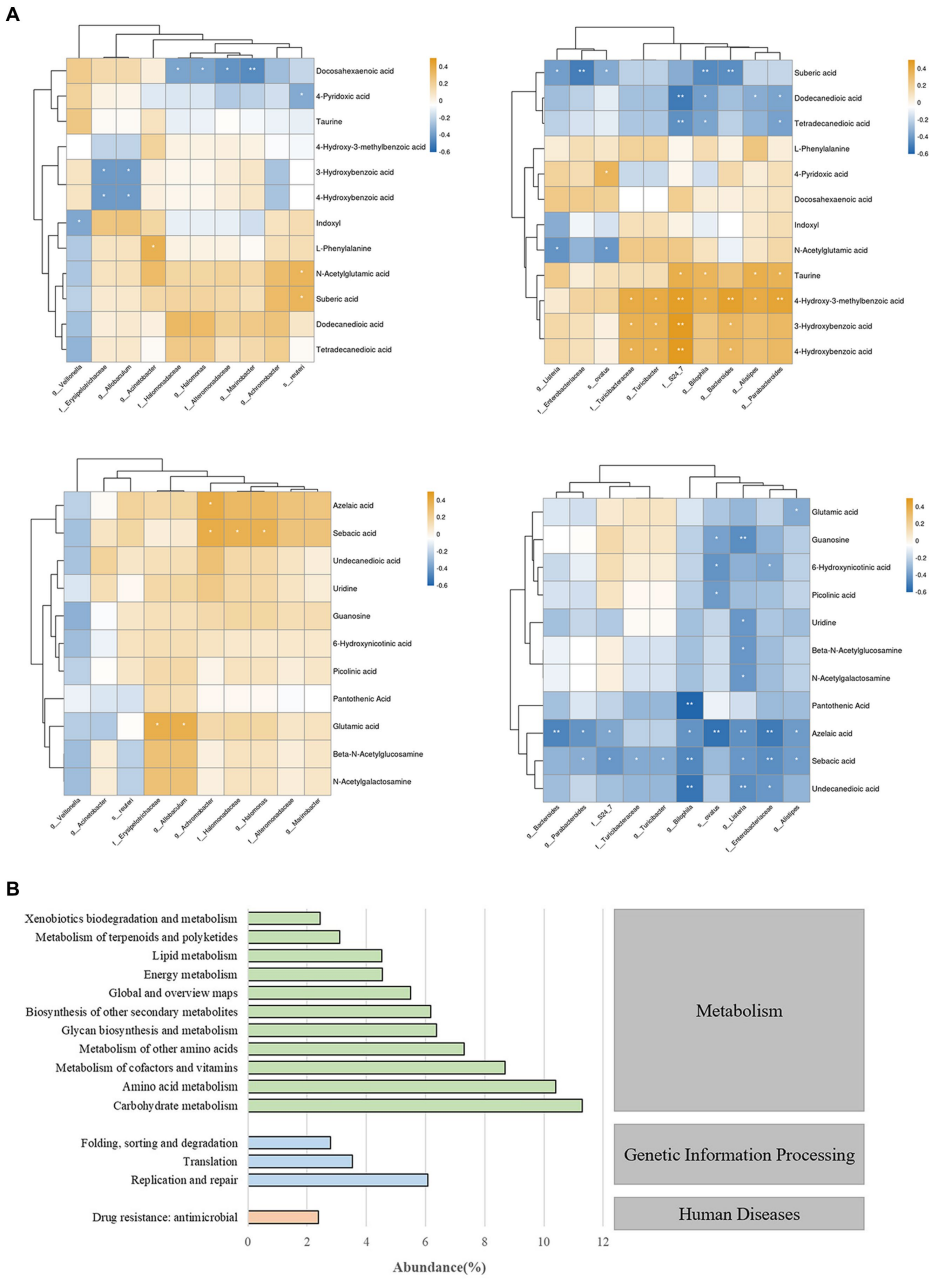
**(A)** Correlation analysis chart of gut microbiota and fecal metabolites. ^*^ indicates *p* < 0.05; ^**^ indicates *p* < 0.01. **(B)** Prediction and analysis of gut microbiota function in mice.

To investigate the relationship between differentially abundant metabolites and differentially abundant species in gut microbiota, we performed correlation clustering analysis, as shown in Figure 7A. *Enterobacteriaceae* and *Listeria* were significantly negatively correlated with undecanedioic, sebacic acid, azelacic acid, sebaric acid, guanosine, and uridine. While *bilophila* was positively correlated with taurine, 4-Hydroxy-3-methylbenzoic acid and negtively correlated with various fatty acids levels. On the other hand, the enrichment of several bacterial taxa may affect the levels of 3-Hydroxybenzoic acid, azelaic acid and sebacic acid.

To gain a deeper understanding of the roles and functions of gut microbiota in mice, we conducted a comprehensive functional prediction analysis (Figure 7B). Our findings revealed that these gut microbiota are widely involved in various metabolic activities, including carbohydrate metabolism, amino acid metabolism, metabolism of cofactors and vitamins etc. These results reveal that gut microbiota play a significant role in maintaining the host’s homeostasis and health, and this impact is closely related to their metabolic activities.

## 4. Discussion

Nowadays, there is a widespread and profound understanding of the role of natural products in disease intervention. Due to the large number and variety of small molecules, it is still necessary to seek more efficient and convenient methods to explore potential effective small molecule substances. Network pharmacology, which utilizes multiple databases and integrates various bioinformatics analyses, can effectively aid in addressing these issues (Wang et al., 2021).

In this study, we first utilized network pharmacology techniques to analyze the active ingredients in *Radix Puerariae*, and based on GO and PPI analysis, core protein targets for ulcerative colitis were identified, and molecular docking was performed between active ingredients and targets, resulting in the identification of puerarin as the most promising potential small molecule substance. We confirmed that it can effectively improve the pathological symptoms of DSS-induced colitis in mice, including restoring mouse weight and colon length, inhibiting intestinal tissue damage, and reducing DAI scores. In our experiments, the decrease in concentrations of IL-1β, IL-6 and TNF-α indicated that puerarin has an inhibitory effect on intestinal inflammation. Previous studies have also shown that puerarin can promote goblet cell secretion of mucin to rebuild the mucus layer, forming a barrier between bacteria and epithelial cells to alleviate colitis (Wu et al., 2021).

The microbial community inhabiting the gut plays an essential role in maintaining intestinal health. This study revealed that UC mice exhibited a reduction in *Firmicutes* levels and an increase in *Bacteroidetes* levels within their gut microbiota. The change in the F/B ratio is considered a manifestation of gut dysbiosis. *Firmicutes* are known to produce more energy from equivalent amounts of food compared to other bacterial groups, thus their reduction may lead to weight loss observed in colitis mice. Conversely, *Bacteroidetes* exhibit pro-inflammatory properties due to their lipopolysaccharides (Shen et al., 2018). The observed decrease in F/B ratio is closely associated with inflammatory bowel disease (Stojanov et al., 2020).

In DSS-induced colitis mice, *Alistipes*, *P. copri*, *Enterobacteriaceae*, *Veillonella* and other bacterial groups are significantly enriched. Studies have shown that they can enhance susceptibility to colorectal cancer and arthritis by interfering with IL-6/STAT3 signaling pathway and bile acid metabolism (David et al., 2014; Hofer, 2014; Parker et al., 2020). The differential bacteria *Listeria* has a higher infectivity for immunodeficient patients and also has a higher pathogenicity in IBD (Azimi et al., 2018). Recent studies have also suggested that *Enterobacteriaceae* can use mucosal inflammation to gain a competitive advantage in obtaining energy among symbiotic bacteria, thereby enriching and exacerbating the development of colitis (Garrett et al., 2010). In addition, our correlation analysis between inflammatory factors and differential species also shows a highly positive correlation between these inflammatory pathogenic bacteria and the concentrations of IL-1β, IL-6 and TNF-α. All of these indicate that the enrichment of these pathogenic and pro-inflammatory microorganisms as significant differential species in the UC group is a key factor leading to dysbiosis in the gut microbiota of colitis mice.

Compared with the UC group, after treatment with puerarin, the F/B ratio was restored, inflammation subsided, and the dominant pro-inflammatory bacterial groups were no longer present. Furthermore, it further reversed the significant changes in abundance of bacterial groups in the model group, such as increasing the level of *Desulfovibrionaceae*, which is also a type of bacteria that performs well in anti-inflammatory and anti-obesity aspects (Popli et al., 2022).

For better understanding the role of these bacteria in the gut and their interrelationships, we first conducted Spearman correlation analysis on the microbiota of mice in the model group and the puerarin intervention group. We found that the bacteria formed a tightly connected network. Interestingly, some bacteria that significantly decreased in disease, such as *Allobaculum*, showed significant negative correlations with pathogenic bacteria such as *Alistipes* and *Listeria*. This suggests that in a diseased state, bacteria may resist the growth of other bacterial species by taking advantage of their ecological niche, ultimately resulting in increased abundance, while bacteria negatively correlated with them decrease in abundance. In addition, using PICRUSt to predict the function of the mice microbiota, we found that the top-ranking abundance were involved in various metabolic activities, such as carbohydrate metabolism, amino acid metabolism, and metabolism of cofactors and vitamins. These results all indicate that the gut microbiota, as a special type of organism within the body, has a crucial impact on the host due to its close interrelationships and rich metabolic activities.

Recently, there have been continuous discussions about how metabolites from gut microbiota affect immune homeostasis, host energy metabolism and intestinal mucosal integrity in inflammatory bowel disease (Lavelle and Sokol, 2020). Our previous study found that indole-3-propionic acid derived from the tryptophan pathway can enhance mucous barrier by increasing secretion of mucin (MUC2 and MUC4) and goblet cell products (Li J. et al., 2021). Song et al. also reported that 3-oxo-deoxycholic acid, a bile acid metabolite, can regulate Foxp3 Treg cell homeostasis in the intestinal lamina propria to exert anti-inflammatory properties (Song et al., 2020). Although the oral bioavailability of screened puerarin did not reach ideal standards, recent reports suggest that these polyphenolic small molecules can remain in the intestinal lumen and participate in gut microbiota metabolism to exert their functions (Luca et al., 2020).

In this experiment, it was found that levels of fecal metabolites such as glutamate and 4-pyridoxic acid decreased while taurine levels significantly increased in UC mice. Taurine is known to activate NLRP6 inflammasomes leading to inflammatory reactions in the intestinal mucosal layer and activation of vitamin B6 degradation pathways during cellular immune responses (Levy et al., 2015). However, no changes were observed in these metabolic pathways after treatment with puerarin. Additionally, tryptophan was not detected but its secondary metabolite indole derivative significantly increased in DSS-induced colitis mice.

Further metabolic pathway analysis of UC mice showed significant changes in the tryptophan metabolism pathway. In recent studies, it has similarly been found that DSS-induced colitis disrupts tryptophan/kynurenine metabolism through the regulation of the rate-limiting enzyme IDO1, and this phenomenon is closely associated with alterations in the gut microbiota (Zhao et al., 2023). Differential species analysis of fecal microbiota also revealed a significant increase in the abundance of the *Bacteroides* and *Enterobacteriaceae* in the UC group. Previous reports have suggested that *Bacteroides* is involved in many important metabolic activities in the colon, including carbohydrate fermentation, nitrogenous substance utilization, and biotransformation of bile acids and other steroids. Some species of *Bacteroides* can produce indole-3-lactic acid, indole-3-acetic acid, and tryptamine (Liu Y. et al., 2023; Nie et al., 2023). *Enterobacteriaceae* have also been reported to have the ability to metabolize tryptophan *in vivo* (Zhang J. et al., 2021). These findings suggest that there may be an imbalance and excessive utilization of tryptophan in the UC mice. Therefore, metabolic disorders such as the indole pathway, urea cycle, glutamate, vitamins, and taurine may exacerbate the development of intestinal inflammation in colitis mice.

After administration of puerarin, the abundance of the *Bacteroides* in the gut was found to be decreased, and the metabolic levels of 3-HAA, picolinic acid, and niacin metabolites were reversed. Among them, 3-HAA is a type of anti-inflammatory tryptophan metabolite, an increase in its concentration can inhibit the development of inflammation in collagen-induced arthritis models. Other studies have shown that 3-HAA can induce apoptosis in liver cancer cells as a ligand-activated transcription factor YY1 and has anti-cancer effects (Kolodziej, 2013; Shi et al., 2021). Puerarin may have an inhibitory effect on intestinal inflammation by regulating the tryptophan metabolism pathway. Yang et al. found that the active ingredient, theabrownin, in Fu Brick Tea can effectively correct intestinal damage in UC mice and promote the secretion of indole derivatives, which are downstream products of the tryptophan metabolic pathway (Yang et al., 2023). This effective improvement of the disease by alleviating the dysregulation of tryptophan metabolism in the microbiota was also observed after turmeric polysaccharides intervention (Yang et al., 2021).

In our results, the differential bacterial groups *Rhodobacteraceae* and *Halomonadaceae* after puerarin treatment contain enzymes that break down tryptophan into kynurenine and downstream metabolites (Vujkovic-Cvijin et al., 2013). Therefore, we speculate that the improvement effect of puerarin on UC may be partially achieved by restoring the function of gut microbiota and thus restoring tryptophan metabolism.

In addition, we correlated differential species and metabolites and found that *Enterobacteriaceae* and *Listeria* were significantly and negatively correlated with undecanedioic, sebacic acid, azelacic acid, sebaric acid, guanosine, and uridine, which are pathogenic bacteria that dominate under disease conditions. After treatment, we also observed a significant increase in purine metabolites such as uridine and guanosine, which have been reported in previous studies to activate the PPARγ signaling pathway in human colonic epithelial cells and restore mucosal barrier function (Li D. et al., 2021). These results well demonstrate that puerarin alleviates DSS-induced colitis by restoring the abundance and metabolic advantage of specific bacterial communities in mice.

Overall, our research indicates that puerarin intervention effectively reduced pathological damage and inflammatory status in DSS-induced colitis mice, and partially restored gut microbiota and metabolic disorders. Our study expands on the potential effects of puerarin in improving intestinal inflammation based on previous research, providing data support for the exploration and development of such natural small molecule substances in the future.

## Data availability statement

The datasets presented in this study can be found in online repositories. The names of the repository/repositories and accession number(s) can be found in the article/Supplementary material.

## Ethics statement

The animal study was approved by the Animal Experimentation Ethical Committee of Nanchang University (License number: SYXK 2021-0001). The study was conducted in accordance with the local legislation and institutional requirements.

## Author contributions

YZ: Conceptualization, Data curation, Formal analysis, Investigation, Methodology, Software, Visualization, Writing – original draft. WD: Conceptualization, Data curation, Methodology, Visualization, Writing – original draft. YW: Methodology, Software, Supervision, Writing – original draft. TC: Data curation, Formal analysis, Investigation, Writing – original draft. ZR: Funding acquisition, Resources, Supervision, Validation, Writing – review & editing, Project administration.

## Footnotes

^1^ https://old.tcmsp-e.com/tcmsp.php

^2^ https://go.drugbank.com/

^3^ https://www.uniprot.org/

^4^ https://www.disgenet.org/home/

^5^ https://www.genecards.org/

^6^ https://string-db.org/

^7^ https://DAVID.ncifcrf.gov/

^8^ https://pubchem.ncbi.nlm.nih.gov/

^9^ https://www.rcsb.org/

^10^ http://www.metaboanalyst.ca
