# Supplementary material for: Puerarin alleviates inflammation and pathological damage in colitis mice by regulating metabolism and gut microbiota
Source: Front Microbiol. 2023 Oct 16;14:1279029. doi: 10.3389/fmicb.2023.1279029 (PMC10614640; doi:10.3389/fmicb.2023.1279029)
Supplement: Supplementary file 1 [file Data_Sheet_1.docx]

Supplementary Material

Puerarin alleviates inflammation and pathological damage in colitis mice by regulating metabolism and gut microbiota

Yixin Zou^1^, Wenjiao Ding^1^, You Wu^1^, Tingting Chen^1^, Zheng Ruan^1^*

*** Correspondence:** Zheng Ruan: [ruanzheng@ncu.edu.cn](mailto:ruanzheng@ncu.edu.cn)

# Supplementary Tables

**Table S1** Disease activity index (DAI).

| Score | Stool consistency | Fecal hemorrhage | Weight loss (%) |
| --- | --- | --- | --- |
| 0 | Normal | Normal | <2 |
| 1 | Mild soft stool | Brown | 2-5 |
| 2 | Very soft stool | Red | 5-10 |
| 3 | watery stool | Gross bleedings | 10-15 |
| 4 | / | / | ≥15 |

**Table S2** Information of active ingredients in *Radix Puerariae.*

| MOL ID | Name | OB | DL |
| --- | --- | --- | --- |
| MOL000392 | formononetin | 69.67 | 0.21 |
| MOL002959 | 3'-Methoxydaidzein | 48.57 | 0.24 |
| MOL000358 | beta-sitosterol | 36.91 | 0.75 |
| MOL012297 | puerarin | 24.03 | 0.69 |
| MOL000390 | daidzein | 19.44 | 0.19 |
| MOL000481 | genistein | 17.93 | 0.21 |
| MOL009720 | daidzin | 14.32 | 0.73 |
| MOL000391 | Ononin | 11.52 | 0.78 |

**Table S3** Docking Information for compounds and Target Molecules.

| Ingredients | Chemical Formula | -CDOCKER INTERACTION ENERGY | Target |
| --- | --- | --- | --- |
| puerarin | C_21_H_20_O_9_ | 65.376 | TNF |
| puerarin | C_21_H_20_O_9_ | 65.183 | TNF |
| genistein | C_15_H_10_O_5_ | 49.633 | TNF |
| genistein | C_15_H_10_O_5_ | 48.896 | TNF |
| formononetin | C_16_H_12_O_4_ | 45.979 | TNF |
| formononetin | C_16_H_12_O_4_ | 45.974 | TNF |
| daidzein | C_15_H_10_O_4_ | 45.823 | TNF |
| daidzein | C_15_H_10_O_4_ | 45.117 | TNF |
| puerarin | C_21_H_20_O_9_ | 37.515 | IL-1B |
| genistein | C_15_H_10_O_5_ | 36.315 | IL-1B |
| daidzein | C_15_H_10_O_4_ | 35.567 | IL-1B |
| puerarin | C_21_H_20_O_9_ | 34.55 | IL-1B |
| genistein | C_15_H_10_O_5_ | 33.848 | IL-1B |
| formononetin | C_16_H_12_O_4_ | 30.302 | IL-1B |
| daidzein | C_15_H_10_O_4_ | 30.149 | IL-1B |
| formononetin | C_16_H_12_O_4_ | 29.025 | IL-1B |
| puerarin | C_21_H_20_O_9_ | 51.831 | IL10 |
| puerarin | C_21_H_20_O_9_ | 51.453 | IL10 |
| daidzein | C_15_H_10_O_4_ | 50.186 | IL10 |
| daidzein | C_15_H_10_O_4_ | 49.425 | IL10 |
| formononetin | C_16_H_12_O_4_ | 43.679 | IL10 |
| genistein | C_15_H_10_O_5_ | 40.852 | IL10 |
| genistein | C_15_H_10_O_5_ | 40.733 | IL10 |
| formononetin | C_16_H_12_O_4_ | 40.363 | IL10 |

**Table S4** Changes of metabolites among UC and Control group.

| Number | Name | VIP | FC | Change |
| --- | --- | --- | --- | --- |
| 1 | Azelaic acid | 2.01 | 0.42 | down |
| 2 | 3-Hydroxybenzoic acid | 1.86 | 4.12 | up |
| 3 | 4-Hydroxybenzoic acid | 1.86 | 4.12 | up |
| 4 | Sebacic acid | 1.84 | 0.38 | down |
| 5 | Suberic acid | 1.84 | 0.48 | down |
| 6 | 3-Hydroxyanthranilic acid | 1.83 | 0.36 | down |
| 7 | Docosahexaenoic acid | 1.74 | 2.63 | up |
| 8 | Undecanedioic acid | 1.66 | 0.44 | down |
| 9 | Dodecanedioic acid | 1.63 | 0.34 | down |
| 10 | Uridine | 1.62 | 0.18 | down |
| 11 | Tetradecanedioic acid | 1.56 | 0.36 | down |
| 12 | Indolelactic acid | 1.52 | 35.08 | up |
| 13 | Pantothenic Acid | 1.52 | 0.59 | down |
| 14 | 4-Hydroxy-3-methylbenzoic acid | 1.50 | 6.40 | up |
| 15 | Guanosine | 1.49 | 0.45 | down |
| 16 | Glutamic acid | 1.40 | 0.42 | down |
| 17 | L-Phenylalanine | 1.34 | 9.48 | up |
| 18 | Indoxyl | 1.30 | 0.56 | down |
| 19 | 4-Pyridoxic acid | 1.29 | 0.72 | down |
| 20 | 6-Hydroxynicotinic acid | 1.22 | 0.35 | down |
| 21 | N-Acetylgalactosamine | 1.15 | 0.79 | down |
| 22 | Beta-N-Acetylglucosamine | 1.15 | 0.79 | down |
| 23 | N-Acetylglutamic acid | 1.14 | 0.22 | down |
| 24 | Taurine | 1.10 | 8.02 | up |
| 25 | Picolinic acid | 1.07 | 0.41 | down |
| 26 | L-Methionine | 1.03 | 2.95 | up |

**Table S5** Changes of metabolites among UC and PUE group.

| Number | Name | VIP | FC | Change |
| --- | --- | --- | --- | --- |
| 1 | N-Acetylglutamic acid | 1.93 | 9.17 | up |
| 2 | Guanosine | 2.01 | 7.57 | up |
| 3 | Picolinic acid | 1.71 | 4.46 | up |
| 4 | 6-Hydroxynicotinic acid | 1.68 | 3.66 | up |
| 5 | 3-Hydroxyanthranilic acid | 2.18 | 3.20 | up |
| 6 | Tetradecanedioic acid | 1.18 | 1.92 | up |
| 7 | 4-Hydroxybenzoic acid | 2.31 | 1.90 | up |
| 8 | 3-Hydroxybenzoic acid | 2.31 | 1.90 | up |
| 9 | Dodecanedioic acid | 1.24 | 1.76 | up |
| 10 | Undecanedioic acid | 1.33 | 1.71 | up |
| 11 | Sebacic acid | 1.37 | 1.66 | up |
| 12 | N-Acetylgalactosamine | 1.46 | 1.63 | up |
| 13 | Beta-N-Acetylglucosamine | 1.46 | 1.63 | up |
| 14 | Azelaic acid | 1.46 | 1.54 | up |
| 15 | Vaccenic acid | 1.46 | 1.52 | up |
| 16 | Oleic acid | 1.46 | 1.52 | up |
| 17 | Elaidic acid | 1.46 | 1.52 | up |
| 18 | Docosahexaenoic acid | 1.44 | 0.53 | down |

**Table S6** Changes of metabolites among UC and SASP group.

| Number | English name | VIP | FC | Change |
| --- | --- | --- | --- | --- |
| 1 | 3-Aminosalicylic acid | 2.71 | 19452 | up |
| 2 | 3-Hydroxyanthranilic acid | 2.71 | 19452 | up |
| 3 | Uridine | 2.02 | 5.41 | up |
| 4 | L-Methionine | 1.39 | 0.22 | down |
| 5 | Indoxyl | 1.35 | 2.74 | up |
| 6 | N-Acetylglutamic acid | 1.11 | 8.64 | up |
| 7 | Guanosine | 1.11 | 1.83 | up |
| 8 | Glutamic acid | 1.05 | 1.74 | up |

# Supplementary Figures

**Figure S1** PPI analysis of overlapping genes between UC and *Radix Puerariae.*

| 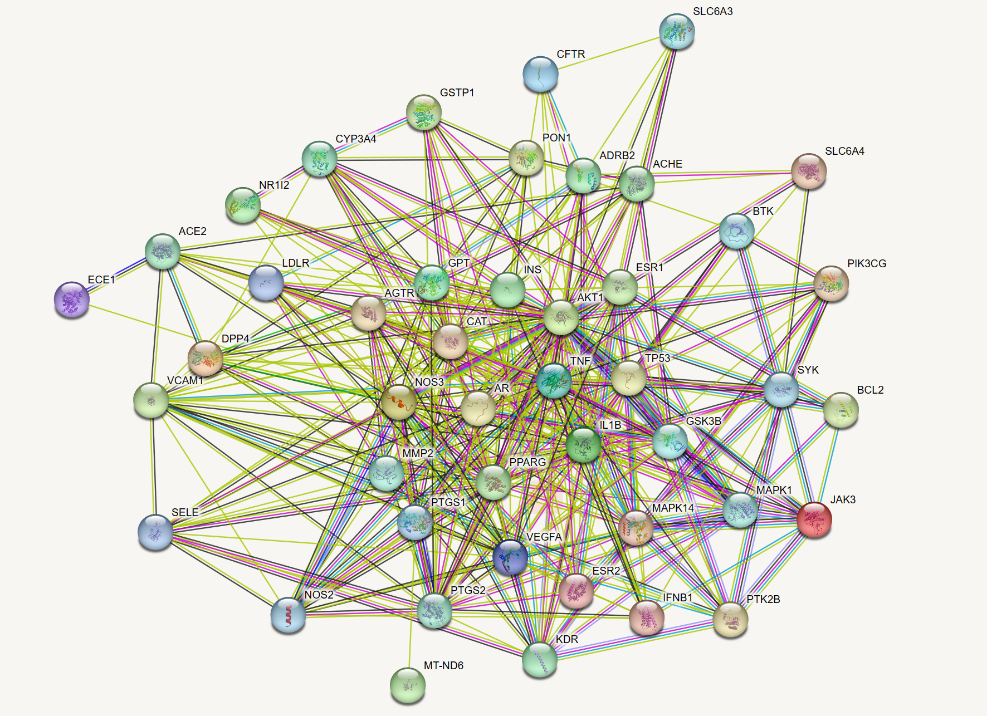 |
| --- |

**Figure S2** GO enrichment analysis of the genes regulated by *Radix Puerariae.*


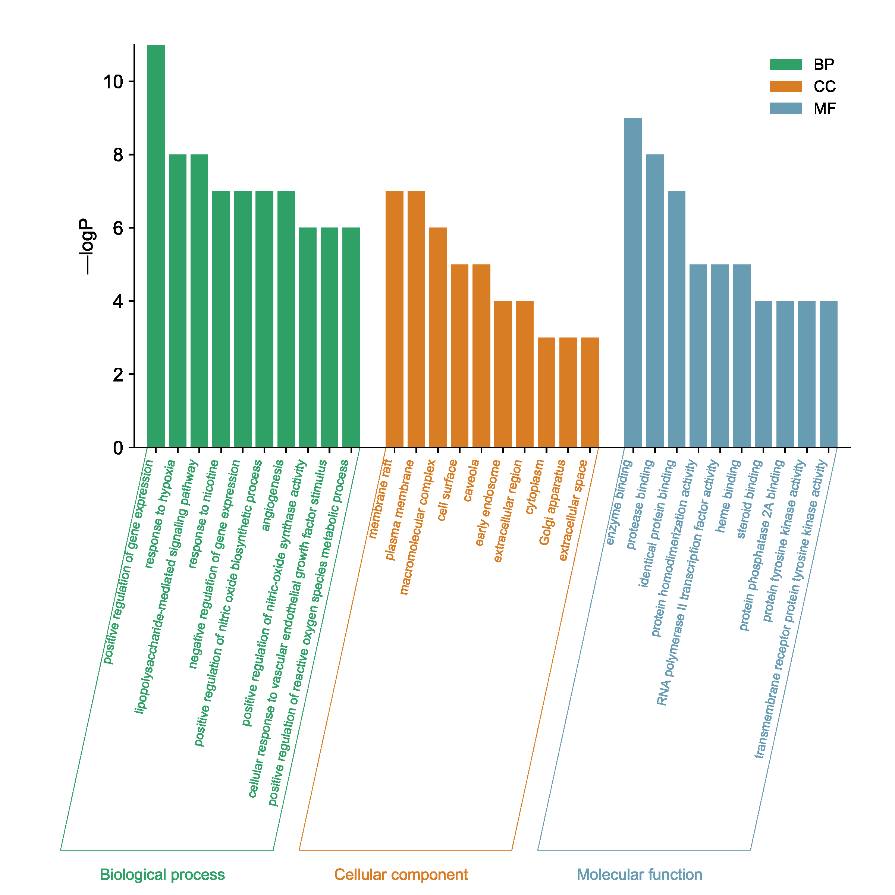


**Figure S3** KEGG pathway analysis of the genes regulated by *Radix Puerariae*.

| 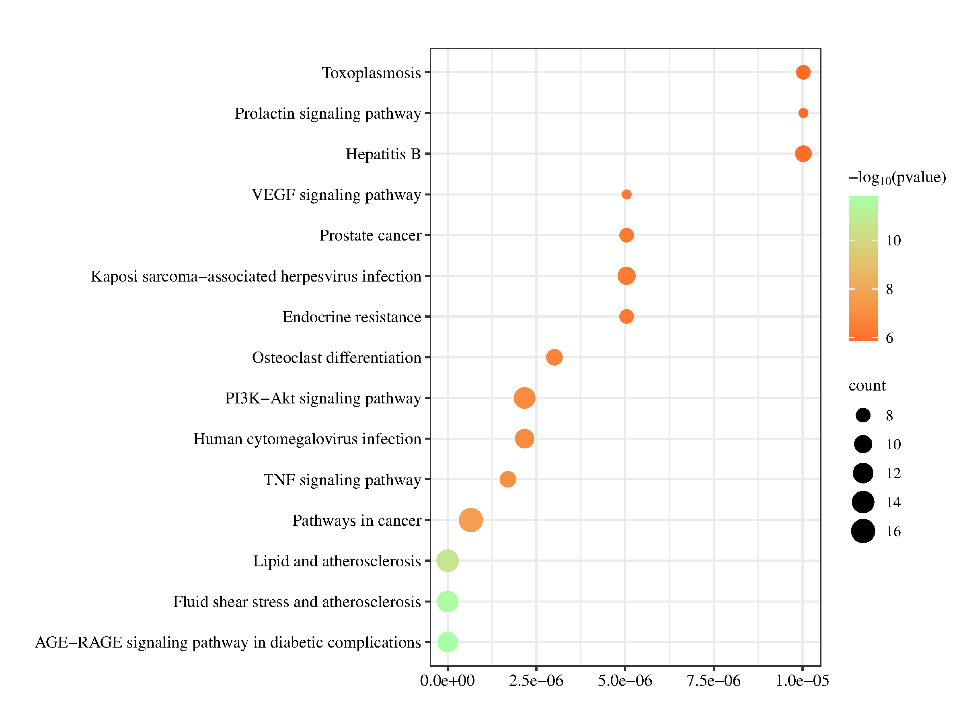 |
| --- |


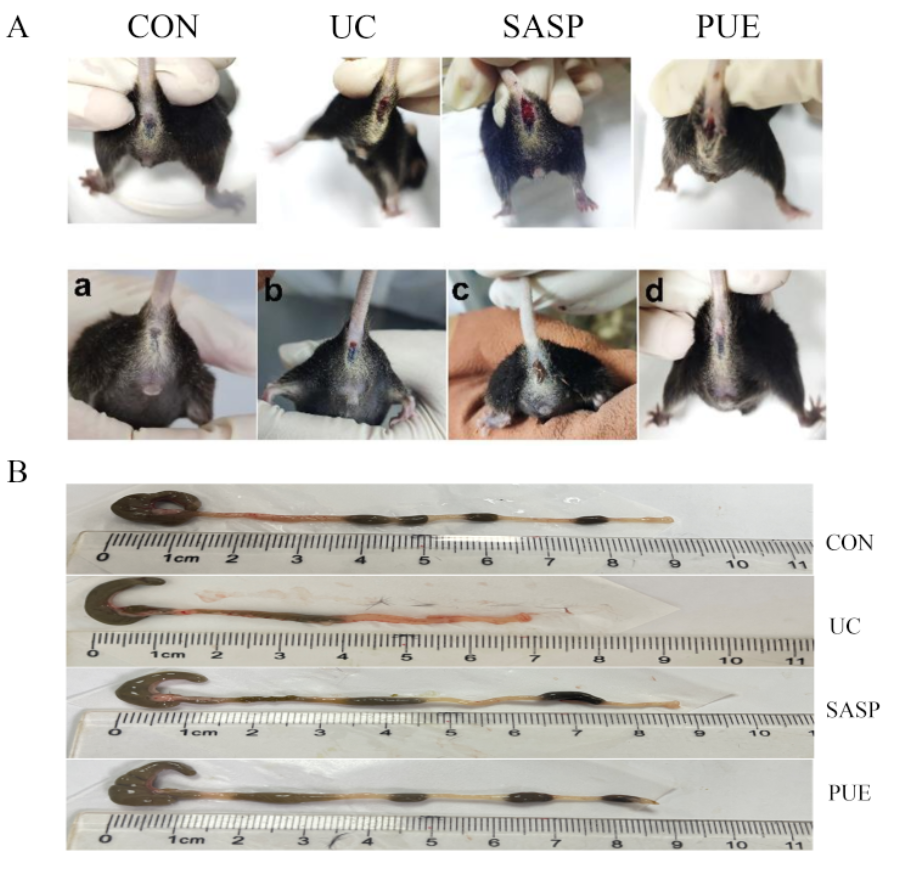
**Figure S4** Colon length and representative colonic morphology of mice in each group.

**Figure S5** Species accumulation curve of microbial in mice feces.

| 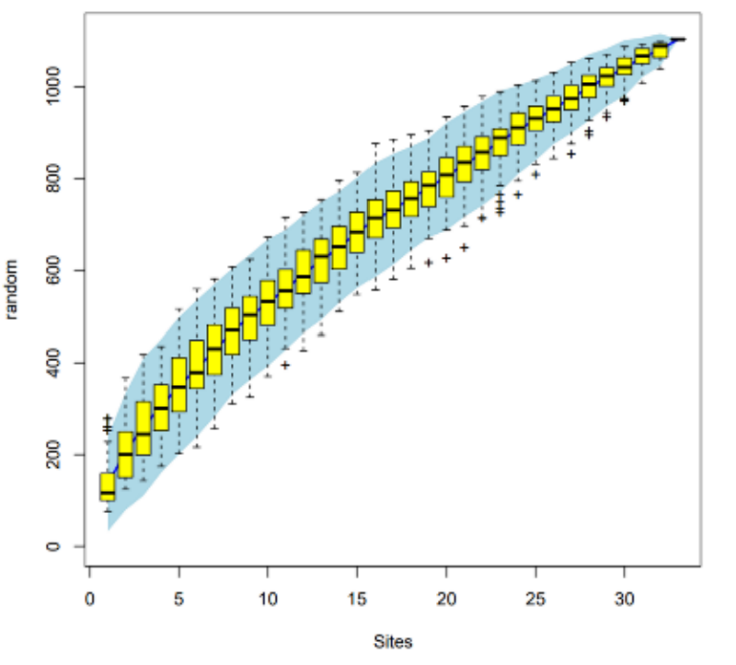 |
| --- |

**
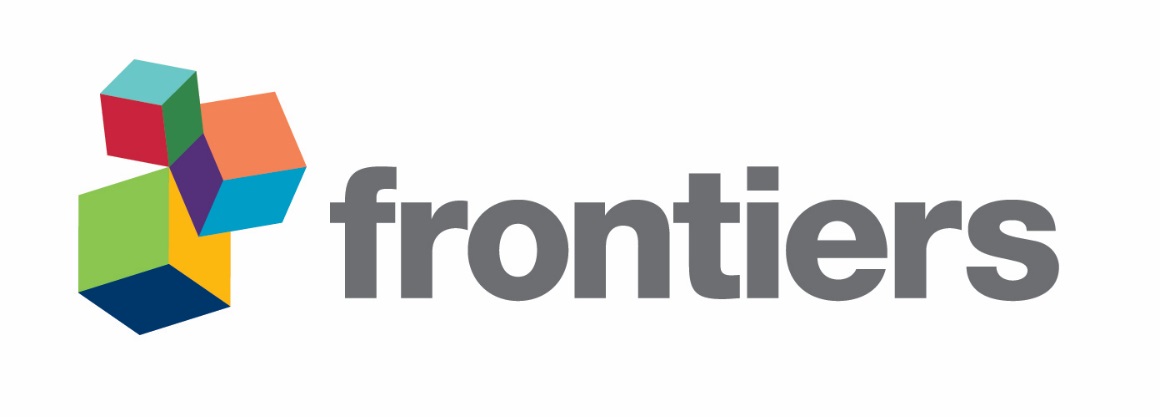
**
